# Climate change induces shifts in coastal Baltic Sea surface water microorganism stress and photosynthesis gene expression

**DOI:** 10.3389/fmicb.2024.1393538

**Published:** 2024-06-07

**Authors:** Laura Seidel, Elias Broman, Magnus Ståhle, Kristofer Bergström, Anders Forsman, Samuel Hylander, Marcelo Ketzer, Mark Dopson

**Affiliations:** ^1^Centre for Ecology and Evolution in Microbial Model Systems (EEMiS), Linnaeus University, Kalmar, Sweden; ^2^Department of Ecology, Environment and Plant Sciences, Stockholm University, Stockholm, Sweden; ^3^Baltic Sea Centre, Stockholm University, Stockholm, Sweden; ^4^Biology and Environmental Science, Linnaeus University, Kalmar, Sweden

**Keywords:** 16S rRNA gene, marine, methanogenesis, methanotrophy, RNA transcripts, stress response

## Abstract

The world’s oceans are challenged by climate change linked warming with typically highly populated coastal areas being particularly susceptible to these effects. Many studies of climate change on the marine environment use large, short-term temperature manipulations that neglect factors such as long-term adaptation and seasonal cycles. In this study, a Baltic Sea ‘heated’ bay influenced by thermal discharge since the 1970s from a nuclear reactor (in relation to an unaffected nearby ‘control’ bay) was used to investigate how elevated temperature impacts surface water microbial communities and activities. 16S rRNA gene amplicon based microbial diversity and population structure showed no difference in alpha diversity in surface water microbial communities, while the beta diversity showed a dissimilarity between the bays. Amplicon sequencing variant relative abundances between the bays showed statistically higher values for, e.g., Ilumatobacteraceae and Burkholderiaceae in the heated and control bays, respectively. RNA transcript-derived activities followed a similar pattern in alpha and beta diversity with no effect on Shannon’s *H* diversity but a significant difference in the beta diversity between the bays. The RNA data further showed more elevated transcript counts assigned to stress related genes in the heated bay that included heat shock protein genes *dnaKJ*, the co-chaperonin *groS*, and the nucleotide exchange factor heat shock protein *grpE*. The RNA data also showed elevated oxidative phosphorylation transcripts in the heated (e.g., *atpHG*) compared to control (e.g., *atpAEFB*) bay. Furthermore, genes related to photosynthesis had generally higher transcript numbers in the control bay, such as photosystem I (*psaAC*) and II genes (*psbABCEH*). These increased stress gene responses in the heated bay will likely have additional cascading effects on marine carbon cycling and ecosystem services.

## Introduction

Climate change related warming is a severe environmental threat affecting all environments (IPCC, 2023) with a recent study suggesting a global warming related temperature rise above pre-industrial levels of 1.7 ± 0.1°C in 2020 (Mcculloch et al., 2024). In addition, the global ocean surface temperature has increased by almost 0.8°C since 1880 (Huang et al., 2017) and between 1971 and 2011 the temperature increased by ~0.11°C per decade (Rhein et al., 2013). Effects on aquatic ecosystems due to an increased water temperature include, e.g., deviations in algal bloom timings, changes in population growth due to increased metabolic rates, invasion of “exotic” species, and increased rate of disease outbreaks (Hoegh-Guldberg and Bruno, 2010). In addition to an increase in temperature, warmer waters contain less soluble oxygen that has resulted in a decrease in global ocean oxygen availability in the last five decades (Schmidtko et al., 2017). This can exacerbate the incidence and extent of oxygen deficient bottom waters, so called ‘dead zones’ (Diaz and Rosenberg, 2008). Environmental changes derived from climate change are therefore important for ecosystem services and functions. However, realistic studies of long-term climate changes on aquatic environments are rare, with many studies being based on short term and/or large changes in temperature, that both lack the influence of annual temperature variations.

Marine phytoplankton are estimated to account for ~45% of all photosynthetic incorporation of CO_2_ into organic matter on the planet (Field et al., 1998), of which a large portion is eventually recycled by bacteria in the water column (Moran et al., 2016). An increase in temperature is typically related to a higher rate of photosynthesis in cyanobacteria (Coles and Jones, 2001). However, this pattern is less obvious in for example, marine Picocyanobacteria that are ubiquitous small photosynthetic bacteria for which an increase in temperature and/or CO_2_ has shown varying results on photosynthesis (i.e., enhanced or stable) depending on the studied genera (Fu et al., 2007).

To investigate the effect of increased water temperature on aquatic communities, recent studies have used a study site located in the south of Sweden close to the city of Oskarshamn near a nuclear power plant (first in production in 1972). The power plant uses Baltic Sea water for cooling inside the reactor chamber (without coming into contact with the radioactivity) and this thermal water discharge has warmed the surface water in a temperature affected bay for more than 50 years by a yearly average of 5°C (Seidel et al., 2022b). This coastal site was used as a case study for predicted climate change effects such as an increased temperature (Pan et al., 2013). In addition, recent publications at this study site compared to a nearby unaffected control bay reveal differences of microbial communities in surface (Seidel et al., 2022b) and deeper sediment depths (Seidel et al., 2023b). The heated bay warming also leads to compression of sediment geochemical layers such that, e.g., the sulfate/methane transition zone occurs closer to the sediment surface along with shifts in seasonal bottom water microbial communities altering, e.g., the cyanobacterial bloom pattern (Seidel et al., 2022a). Furthermore, a laboratory-based thermal gradient study demonstrated the ability of the control and heated bay benthic community’s response to potential future marine heatwaves (Seidel et al., 2023a). The data show a broader thermal tolerance in the heated bay coupled with elevated RNA transcripts related to stress. However, that study lacks an *in situ* surface water investigation of the microbial community responses in the water column.

In this study, microorganisms were captured from surface waters from the temperature affected heated bay and the unaffected nearby control bay and their nucleic acids extracted and sequenced. The aim of this study was to investigate differences in the community structure of microbial communities and their RNA transcript-based activities in surface waters of the two bays. The results will give additional information regarding coastal Baltic Sea microbial communities in relation to potential future climate change warming related environmental changes.

## Materials and methods

### Field sampling

**Figure 1:**
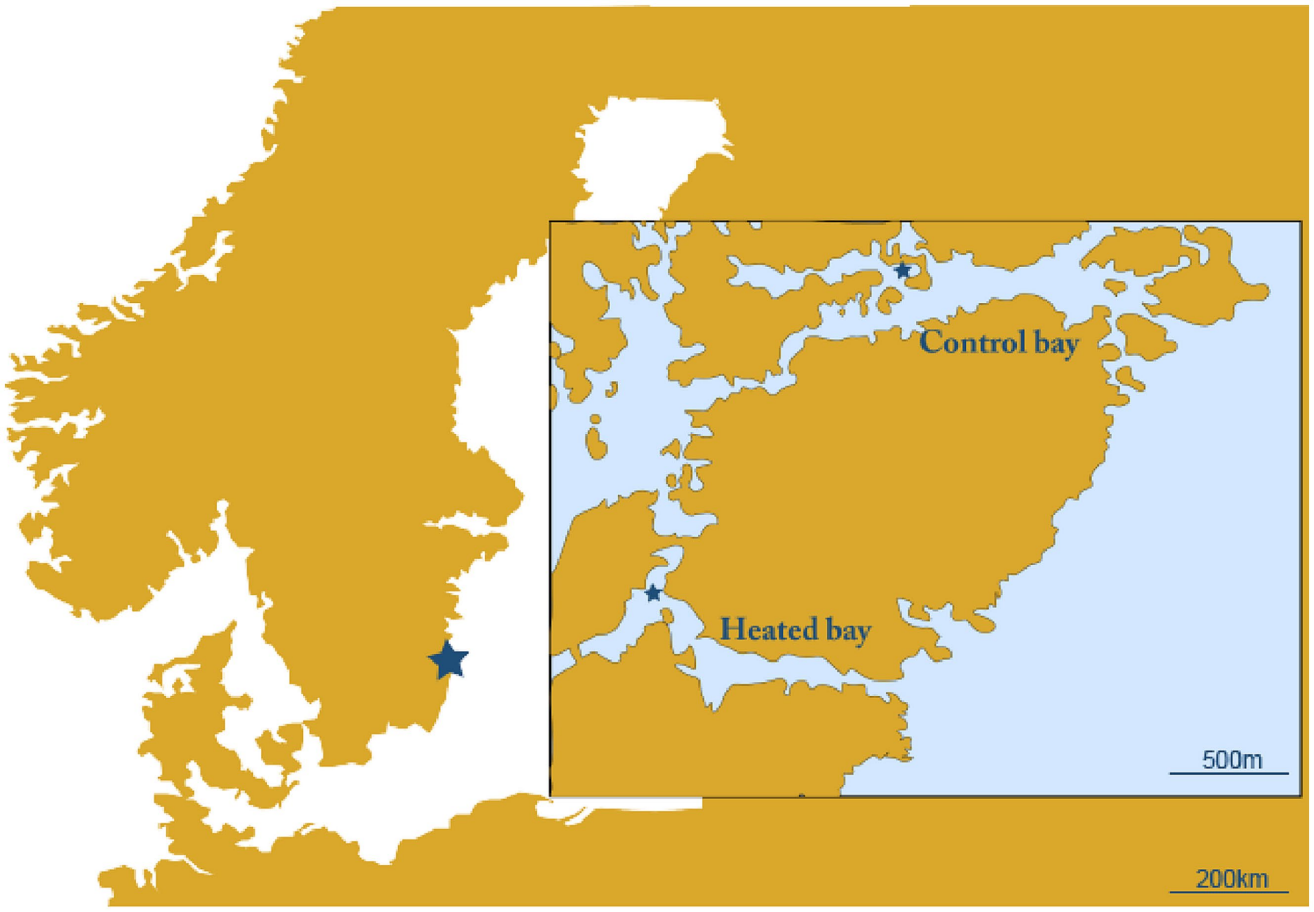
Map of the sampling site. Shown are the heated bay and unconnected control bay with the sampling sites marked with a star.

Two naturally fluctuating coastal systems were used that were chosen based upon their proximity to each other. The first was an enclosed bay affected by the warmed discharge water from the Oskarshamn nuclear power plant, Sweden (heated bay; 57.421750 N, 16.666967E) and the second a nearby bay unaffected by the discharge water from the power plant (control bay; 57.433517 N, 16.683700E; Figure 1). The two sites were sampled for surface water on the 16th and 17th April 2018. Temperature, dissolved oxygen, salinity, and pH were measured in the field with sensors (WTW Multiline). From each site, a total of approximately 29 L surface water (~10 cm below surface) was sampled using a 5 L acid-washed container (~1 M HCl, rinsed with deionized water and *in situ* water) and transferred into acid-washed plus surface water rinsed 20 L and 10 L containers. The water in the 20 L container was used for DNA extraction, while the 10 L container was used for RNA extraction and was mixed with 1 L fix solution to stop microbial activity (5% water-saturated phenol in absolute ethanol) (Feike et al., 2012). The sampled water was transferred to the laboratory (~1.5 h at 11°C–16.5°C) where it was stored overnight in climate rooms set to *in situ* temperatures (heated bay: 13.6°C, control bay: 10.4°C).

Cell capture used two types of 142 mm polycarbonate in-line filter holders (Geotech, United States) with different pore-sizes to capture ultra-small bacteria (0.1 μm Omnipore, hydrophilic polytetrafluoroethylene, Merck and 0.2 μm hydrophilic polycarbonate; Isopore, Merck). The filters were sterilized by ethanol and subsequently autoclaved. Cell capture for DNA extraction was via filtering 2 L heated bay water (*n* = 3 for each filter type) and 1–1.4 L water from control bay (*n* = 3 for each filter type) with the differing volumes due to eventual clogging of the filters. In the same manner, filters for RNA extraction were collected after filtering 1 L of water mixed with fix solution (*n* = 3 for each site and filter type; with no clogging). Filters were transferred into sterile 10 mL cryotubes (VWR Collection, VWR) and stored at −80°C until nucleic acids extraction.

### Nucleic acids extraction and sequencing

DNA was extracted from the frozen filters with the DNeasy PowerWater Kit (QIAGEN) while RNA was extracted and DNase treated with the RNeasy PowerWater Kit (QIAGEN) according to the manufacturer’s instructions. RNA was synthesized into cDNA and subsequently purified using the Ovation RNA-SeqSystemV2 (NuGEN) and MinElute Reaction Cleanup kit (QIAGEN), respectively. The quality of extracted nucleic acids was measured on a NanoDrop 2000 spectrophotometer while concentrations were measured on a Qubit 2.0 fluorometer.

16S rRNA gene amplicon sequencing was conducted by amplifying the partial 16S rRNA gene from the extracted DNA as described previously (Broman et al., 2017a,b). In brief, primers 341F and 805R (Herlemann et al., 2011) were used with PCR programs according to Hugerth et al. (2014). 16S rRNA gene amplicon library preparation was conducted according to Lindh et al. (2015) and sequenced on a Illumina MiSeq flow cell with a 2 × 300 bp setup. All community DNA and RNA (i.e., cDNA on two HiSeq 2,500 lanes with a 2 × 125 bp setup) samples were sequenced at the Science for Life Laboratory, Stockholm, Sweden. One of the 0.2 μm cDNA filters from the control bay was not successfully sequenced by the facility and therefore, this sample group only has two replicates. A complete list of sample names, sequence facility IDs, number of reads obtained before and after merging paired-ends etc., is available in Supplementary Table S2.

### Bioinformatics and statistics

The raw 16S rRNA gene amplicon sequencing data were trimmed, denoised, merged, and chimeras removed by using the nf-core-pipeline for amplicon sequencing called ampliseq (v.2.5.0) within Nextflow (v.23.04.1) using the default settings (Straub et al., 2020). In brief, the forward sequences were trimmed at 269 bp, while the reverse were trimmed at 209 bp and the double removal of the primer sequences were set to true to make sure all primers were removed. The Ampliseq pipeline incorporates DADA2 (v.1.22.0) (Callahan et al., 2016) for denoising and quality control of the sequences followed by generation of amplicon sequence variants (ASVs). Taxonomic classification of the ASVs used SBDI-GTDB (Sativa curated 16S GTDB database, R06-RS202-1; FigShare doi: 10.17044/scilifelab.14869077.v3). Unknown cyanobacterial sequences were blasted against the ‘Phytoref’ database (2017-04-04) to reveal potential eukaryotic diatoms. Further data analysis was conducted using R (v.4.3.1) and RStudio (v.2023.06.2) (Team, 2019). Rarefaction curves (Supplementary Figure S1) were calculated using the ‘vegan’ (v.2.6–4) package using the step size set to 20 and suggested that most microbial diversity was covered in the samples from both bays (Oksanen et al., 2019). Principal component analysis (PCA) based on Bray–Curtis dissimilarities was calculated using relative abundances (normalized counts) and the ‘vegdist’ (distance = bray, default settings) function in vegan plus the ‘prcomp’ function (default settings) in stats (v.4.3.1), while a perMANOVA (999 permutations) comparing the communities between the bays was conducted using the ‘adonis2’ (vegan) function. Alpha diversity analysis (Shannon’s *H*) was performed using the normalization method of scaling with ranked subsampling within the SRS package using the default settings (v.0.2.3) (Beule and Karlovsky, 2020). A non-parametric Kruskal–Wallis (‘Kruskal.test’ function, default settings) test to check for significant differences between the bays in Shannon’s *H* diversity used stats (v.4.3.1). Differential abundance analysis of the ASV counts between the two bays was performed using the ALDEx2 package (‘aldex’ function, conditions = bay, default settings, v.1.32.0) (Gloor, 2015). After the analysis, only significant ASVs (based on Benjamin-Hochberg corrected *p*-values) with an effect size greater than 1/−1 were retained.

The metadenovo (v.2.5.1dev, default settings) nf-core-pipeline for metatranscriptomic data within Nextflow (v.23.04.3) was used for the raw RNA sequences. Briefly, trimming and adapter removal was done by using cutadapt (v.3.4) and trimgalore (v.0.6.7), while sequences were filtered using BBduk (v.39.01). *De novo* assembly was performed with Megahit (v.1.2.9) while the metadenovo orf-caller option Prokka (which uses prodigal) (Hyatt et al., 2010) within the Prokka software tool (v.1.14.6) was used as open read frame caller (Seemann, 2014). For functional annotation eggnog within Eggnog-mapper (v.2.1.9) (Cantalapiedra et al., 2021) was chosen while EUKulele (v.2.0.3) (Krinos et al., 2021) has been used to annotate taxonomical classification. For prokaryote annotation, GTDB was used within EUkulele while a second annotation with PhyloDB (default) was used to specify eukaryotic transcripts. After this, the eukaryotic transcripts were filtered out. The final mRNA transcript output was analyzed using R within RStudio and expressed as transcripts per million (TPM). Alpha diversity (Shannon’s *H*) was calculated using TPM values (García-Nieto et al., 2022) and compared to each other using the non-parametric Kruskal–Wallis test. Differential expression analysis was conducted using the DESeq2 (v.1.40.2) package (Love et al., 2014). Before the analysis comparing the two bays, low abundant counts (at least 10 counts) were filtered out that were not detected within the smallest group sample size (here bay; *n =* 6). The ‘DESeq’ function (settings; test = “LRT,” reduced = ~1, sfType = “poscounts,” minReplicatesForReplace = Inf, fitType = “local”) was used as well as the ‘results’ function (settings; contrast = “heated,” “control”). A PCA was done by log-transforming (‘rlog’ function within the DESeq2 package) the transcripts to account for the compositional nature of relative abundance data (Aitchison, 1986) as well as the ‘prcomp’ function within the stats package. All Plots were generated using the ggplot2 (v.3.5.0) package (Wickham, 2016). A full overview of the used code can be found online, see the data availability statement for further information.

## Results

### Field data

Differences in surface and bottom water temperatures and salinities between the bays for multiple sampling times over several years are provided in Supplementary Table S1 [data previously published in Seidel et al. (2022a)]. The surface water temperatures fluctuated less in the heated bay and the differences between the bays were more pronounced in winter compared to summer. In addition, the bottom water salinities were similar in both bays over several years while the surface water differences were more pronounced. At the time of sampling for this study, the heated bay sampling site had a water depth of 3.6 m, similar temperature (~13.6°C) and salinity (~6.5 PSU) between bottom and surface waters, plus 12.8 mg/L oxygen and pH 8.40 in the bottom waters. The control bay site was unaffected by the thermal discharge and therefore, had colder surface and bottom water temperatures (10.4°C and 8.1°C, respectively). This site was located at a depth of 2.8 m, it had a lower salinity in the surface than in the bottom water (3.8 PSU and 6.2 PSU, respectively), an oxygen concentration of 15.7 mg/L, and the pH was 7.93 (Table 1).

**Table 1:** Overview of *in situ* environmental parameters of surface waters in the heated and control bays (both *n =* 1).

	Temperature (°C)	pH	Salinity (PSU)	Oxygen (mg/L)
Control bay	10.4	7.93	3.8	15.67
Heated bay	13.6	8.4	6.6	14.6

### Nucleic acid sequencing data

The Illumina MiSeq 16S rRNA gene sequencing yielded on average 165,783 ± 42,500 reads per sample (one standard deviation shown) with an average of 107,687 ± 44,187 reads after quality control and merging (Supplementary Table S2). Illumina HiSeq 2,500 RNA-seq yielded on average 54 ± 22 million read-pairs per sample. On average 1.09 ± 0.55 million reads per sample were obtained and 14,835 ± 2,472 open read frames were identified (Supplementary Table S2). No statistical differences on 16S rRNA gene taxonomy assignments or RNA transcript counts for annotated gene functions were detected for the different filter pore-sizes (i.e., 0.1 μm and 0.2 μm) in either the 16S rRNA gene amplicon sequencing and metatranscriptome dataset (Supplementary Table S3). Therefore, the samples were treated as replicates.

### Differences in community compositions between the two bays

**Figure 2:**
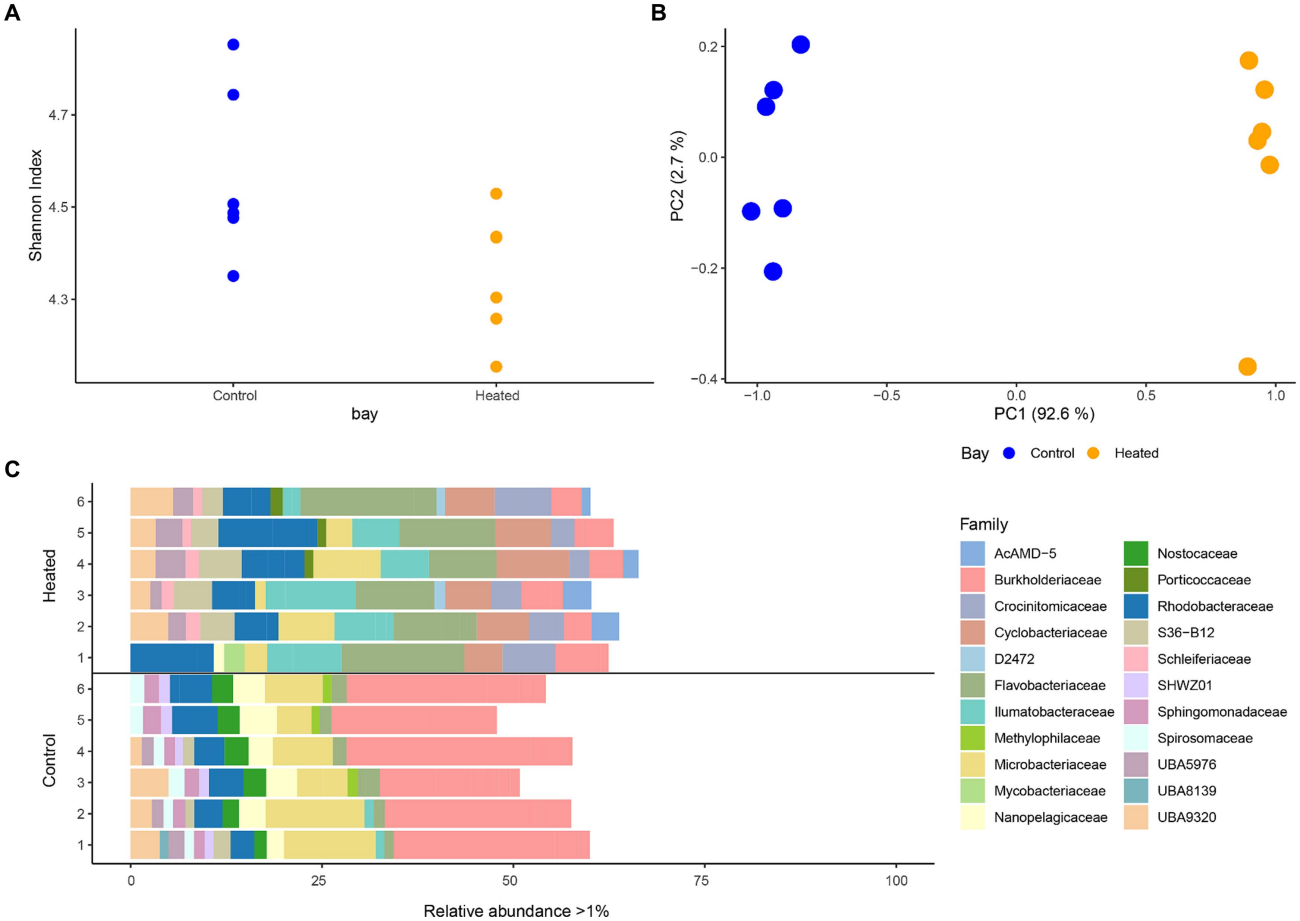
16S rRNA gene amplicon sequencing alpha diversity (mean ± std., *n* = 6; **A**), a PCA based on Bray–Curtis dissimilarities **(B)**, and stacked-bar plot on family level with relative abundance per sample >1% **(C)**. Families with a relative abundance <1% make up the remainder of the abundance. Heated bay is shown in orange while control bay is shown in blue.

Distinct differences were detected between the microbial communities of the heated and control bay’s surface waters with the Shannon’s *H* index in both bays varying across the replicates but showing a slightly higher diversity in the control compared to the heated bay that fell short of the traditional statistical significance level (heated 4.35 ± 0.13, control 4.56 ± 0.18, non-parametric Kruskal–Wallis-Test between bays, *H*_1_ = 3.69, *p* = 0.054; Figure 2). Multivariate PCA analysis using Bray–Curtis dissimilarity on the ASVs showed a statistically significant difference between the two bays (perMANOVA, *F*_1,9_ = 45.4, *p* = 0.003; Figure 2). A clear separation of the two bays microbial communities was observed on the first axis (95.3% explanation) while a small variation between replicates within the bays was shown on the second axis (2.9%; Figure 2).

After filtering phyla with low relative abundances (<0.5% per sample) Actinobacteriota, Bacteroidota, Cyanobacteria, and Proteobacteria were found to be dominant phyla in both bays (Supplementary Figure S2). Comparing the phyla occurrence within each bay showed a higher relative abundance of Proteobacteria in the control bay compared to the heated bay (average relative abundance per bay, heated 17.7 ± 3.6%, control 35.11 ± 3.4%) as well as higher relative abundance of Cyanobacteria (heated 0.7 ± NA %, control 2.5 ± 0.5%). Cyanobacteria was only identified in one replicate of the heated bay samples with a relative abundance >0.5%. In contrast, the Bacteroidota had higher relative abundances in the heated bay compared to the control (heated 31.8 ± 5.1%, control 12.2 ± 2.4%). Differences in microbial communities between the two bays were more distinct on lower taxonomical levels (Figure 2). The top family members with relative abundances >1% included the ecologically diverse Burkholderiaceae (heated bay 4.9 ± 1.2%, control 24.2 ± 3.9%); Microbacteriaceae that are identified in aquatic milieu and in association with, e.g., plants (4.8 ± 3.1, 8.6 ± 3.2); Nanopelagicaeae that can be ultra-small and with streamlined genomes such that they are dependent on a symbiotic partner [1.3 (*n* = 1), 3.7 ± 0.9]; the predominantly aerobic Flavobacteriaceae found in diverse habitats including marine environments where some strains can harvest light energy (12.7 ± 3.4, 1.8 ± 5.5); and the marine Rhodobacteraceae that are involved in carbon and sulfur cycling (8.3 ± 3.0, 4.5 ± 1.1). In addition, Crocinitomicaceae in the Flavobacteriales order that were originally isolated from polar regions (4.7 ± 1.9) and Cyclobacteriaceae that have a range of properties and habitats including temperature and salinity ranges (6.8 ± 1.5) were only present in the heated bay while the heterocytous cyanobacteria Nostocaceae family (2.6 ± 0.5) was only identified in the in the control bay.

**Figure 3:**
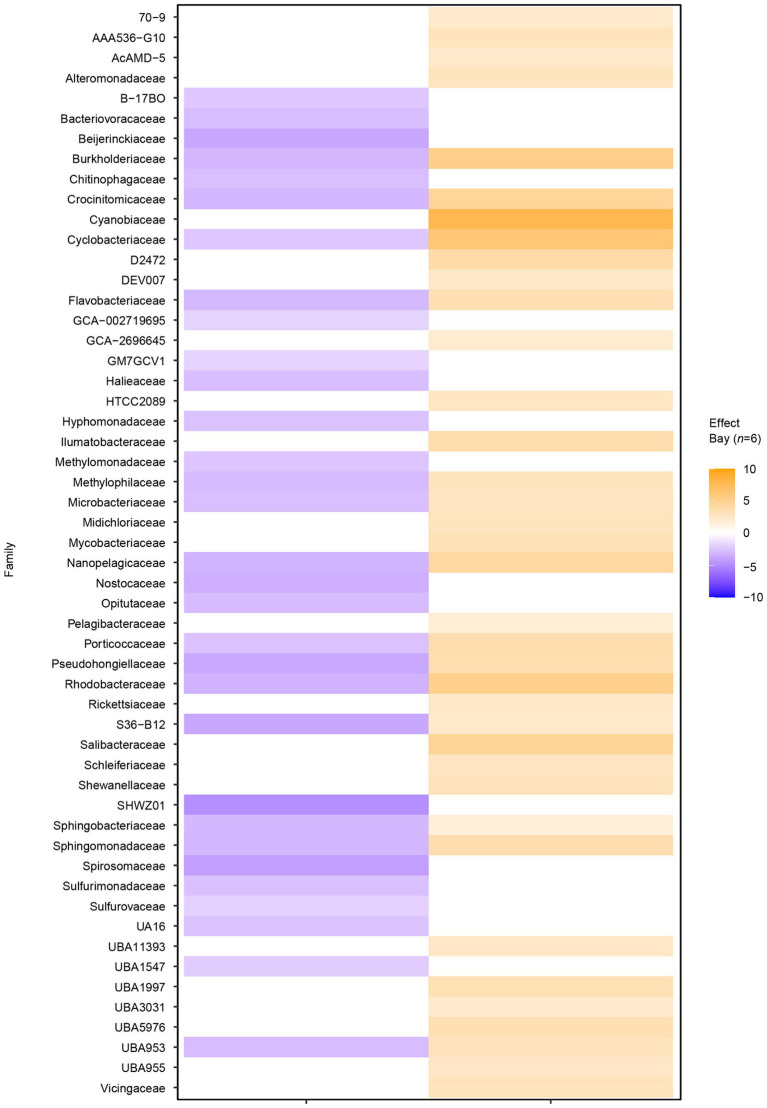
Differential abundance analysis between the two bays on amplicon sequence variant (ASV) level. The figure shows the sum of significant differential abundant ASVs on the level of family. Orange shades indicate higher effect sizes (standardized mean differences between groups) in the heated bay while the blue shades show higher effect sizes in the control bay.

Filtering significant ASV differential abundance (*p* < 0.05) between the bays with at least an effect size >1/−1 showed clear differences on the family level (Figure 3). For example, ASVs with significantly higher abundance in the heated bay included the Ilumatobacteraceae that were originally isolated from desert soil; the chemoorganotrophic Salibacteraceae that require sea salts for growth; along with the candidate families UBA1997 (Rickettsiales bacterium that are obligate intracellular bacteria), UBA3031 (Burkholderiales), UBA5976 (Nanopelagicales), and UBA955 from the Bacteroidetes that comprise up to 30% of marine coastal bacterioplankton. In contrast, ASVs within for example Burkholderiaceae, the photoheterotrophic Halieaceae family of coastal marine Gammaproteobacteria, the methylotrophic Methylomonadaceae that are also implicated in anaerobic oxidation of methane in interaction with other bacteria, Nostocaceae, the sulfur oxidizing Sulfurmonadaceae, and chemolithoautotrophic sulfur oxidizing Sulfurovaceae were significantly higher in abundant in the control bay. Finally, some families had ASVs that were significantly abundant in either one or the other bay, e.g., the methanol (but not methane) oxidizing Methylophilaceae, Rhodobacteraceae, and the UBA953 that is an Opitutae bacteria found in many environments including seawater and marine sediment.

### Differences in RNA transcript levels between bays

**Figure 4:**
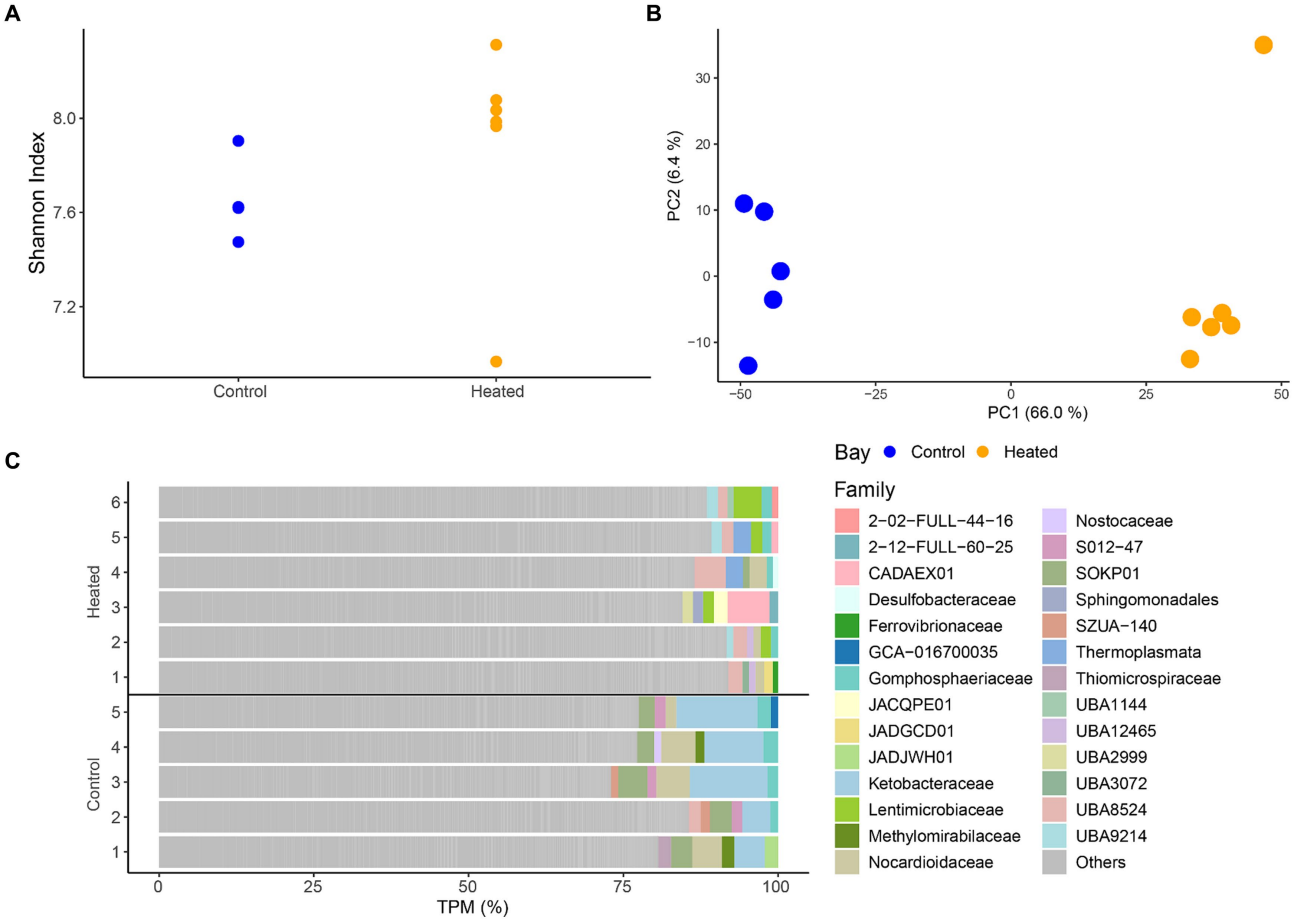
RNA transcript alpha diversity (Shannon’S *H* Index, mean ± std., *n* = 5 to 6) between the two bays **(A)**, a PCA based on log transformed transcripts **(B)**, and the most abundant annotated families per sample with the remainder summarized in ‘Other’ **(C)**. Heated bay is shown in orange while control bay is shown in blue.

Differences in RNA transcripts were observed between the two bays, showing a similar pattern as seen before within the community ASV compositions. Shannon’s *H* diversity was calculated using TPMs as the base for normalization to investigate potential differences between the diversity of transcripts between the bays. The results showed no difference in the heated bay (5.96 ± 1.1) compared to the control bay (6.7 ± 0.3) that had a slightly lower variation [Kruskal–Wallis-Test, insignificant *H*(1) = 0, *p* = 1; Figure 4]. Furthermore, log-transformed transcript counts visualized in a PCA (Figure 4) showed clustering of the samples on the first axis according to bay explaining 66.0% of the variation, while on the second axis a clustering among replicates occurred (explained 6.5% variation).

Taxonomic annotation of the transcripts resulted in 1124 families with the top five within each sample plotted in Figure 4. While there was a large proportion of genes classified as “other,” distinct family groups were present in each bay. The heated bay showed more variation of different families within the higher abundant transcripts with, e.g., Lentimicrobiaceae that is a member of the phylum Bacteroidetes of which the genus *Lentimicrobium* is an anaerobic slow-growing bacterium, UBA8524 from the Bacteroidota, and the acidophilic and mostly thermophilic Thermoplasmata. In contrast, the control bay had higher TPM counts within Ketobacteraceae, UBA3072 (WOR-3), Nocardioidaceae, and Methylomirabilaceae. Similar results were also seen within the differential expression analysis, showing significant higher expression for transcripts related to those families mentioned above, such as Lentimicrobiaceae (heated bay) or Ketobacteraceae (control bay; Supplementary Figure S3). Furthermore, most cyanobacterial related RNA transcripts were higher in the control compared to the heated bay (Supplementary Figure S3).

### Stress related RNA transcripts

**Figure 5:**
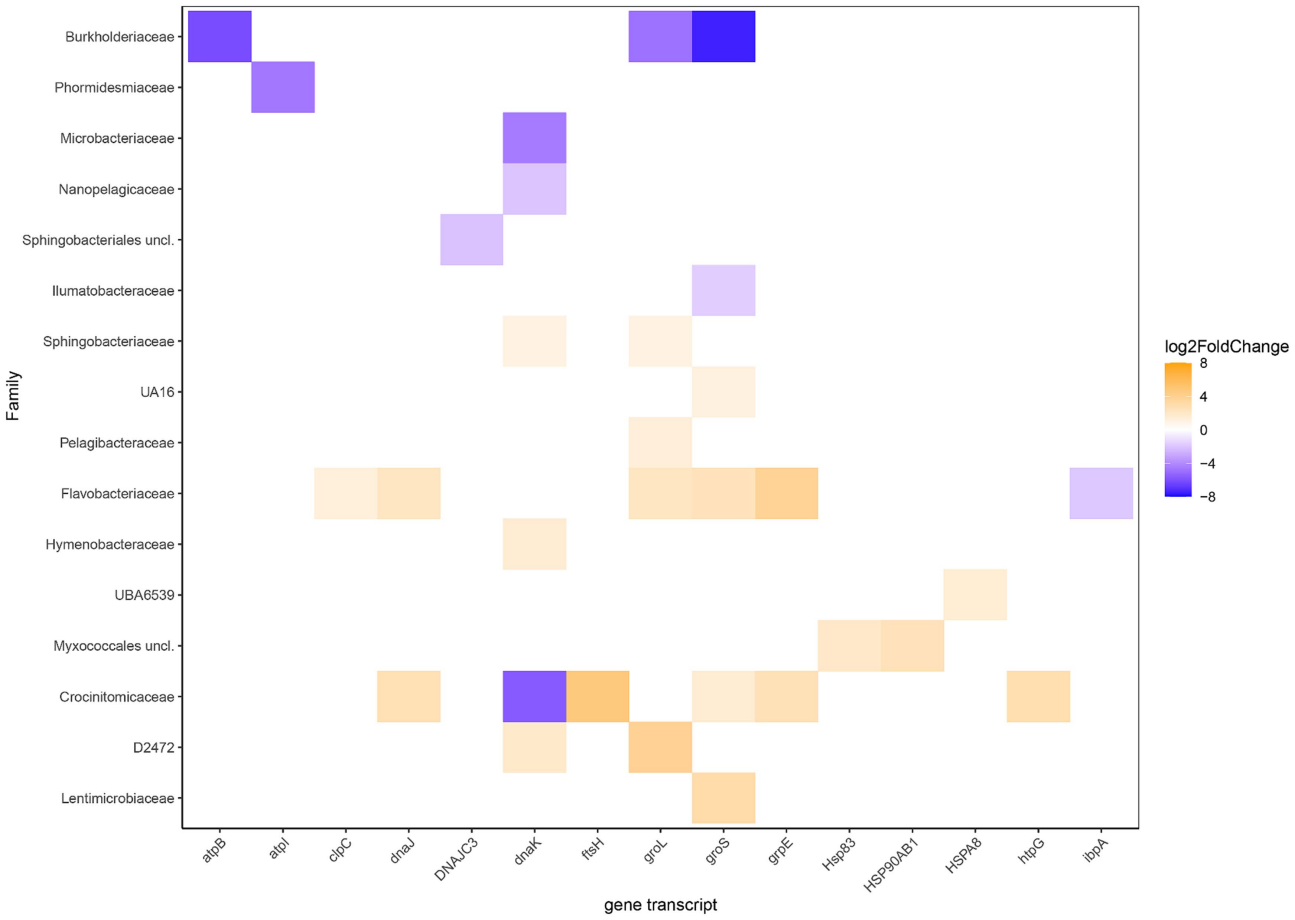
Differential RNA transcript numbers related to stress according to KEGG Orthology and their annotated potential expressed organisms on family level. Blue shows higher RNA transcript numbers expression in the control bay while orange shows higher in the heated bay. The Log_2_FoldChange shows the level of expression of a gene within the tested groups (heated & control) where positive values (orange shades) show higher expression in the heated bay and negative values (blue shades) indicate higher expression in the control bay.

Statistically different RNA transcript numbers showed more transcripts related to stress in the heated compared to the control bay (Figure 5). These transcripts were particularly assigned to the Crocinitomicaceae, Flavobacteriaceae, and Sphingobacteriaceae that are likely adapted to low temperatures and included the heat shock protein HSP70 genes *dnaKJ* [differences between the levels of expression for each gene between the two groups in logarithmic scale (Log_2_FoldChange)] on family level 1.5, 1.3, and 1.1 for Crocinitomicaceae, Flavobacteriaceae, and Sphingobacteriaceae, respectively; the co-chaperonin *groS* that aids in stress-induced mutagenesis (1.6 and 2.4); and nucleotide exchange factor heat shock protein *grpE* (2.8 and 3.8). In addition, other statistically different heated bay stress related RNA transcripts included the heat shock-specific RNA polymerase gene *groL* in family D2472 (3.9) plus the heat shock protein HSP83 (2.1) and HSP90 (2.5) genes in the Myxococcales (unclassified). In contrast, the Burkholderiaceae showed significantly higher stress related gene transcripts in the control bay for *atpB* (−6.1), *groL* (−4.8) that is activated by the heat shock-specific RNA polymerase Eσ^32^, and *groS* (−7.4); the genome streamlined Nanopelagicaceae identified in freshwater for the chaperonin *dnaK* (−2.5); and the Microbacteriaceae for *dnaK* (−4.6).

### Energy metabolism

**Figure 6:**
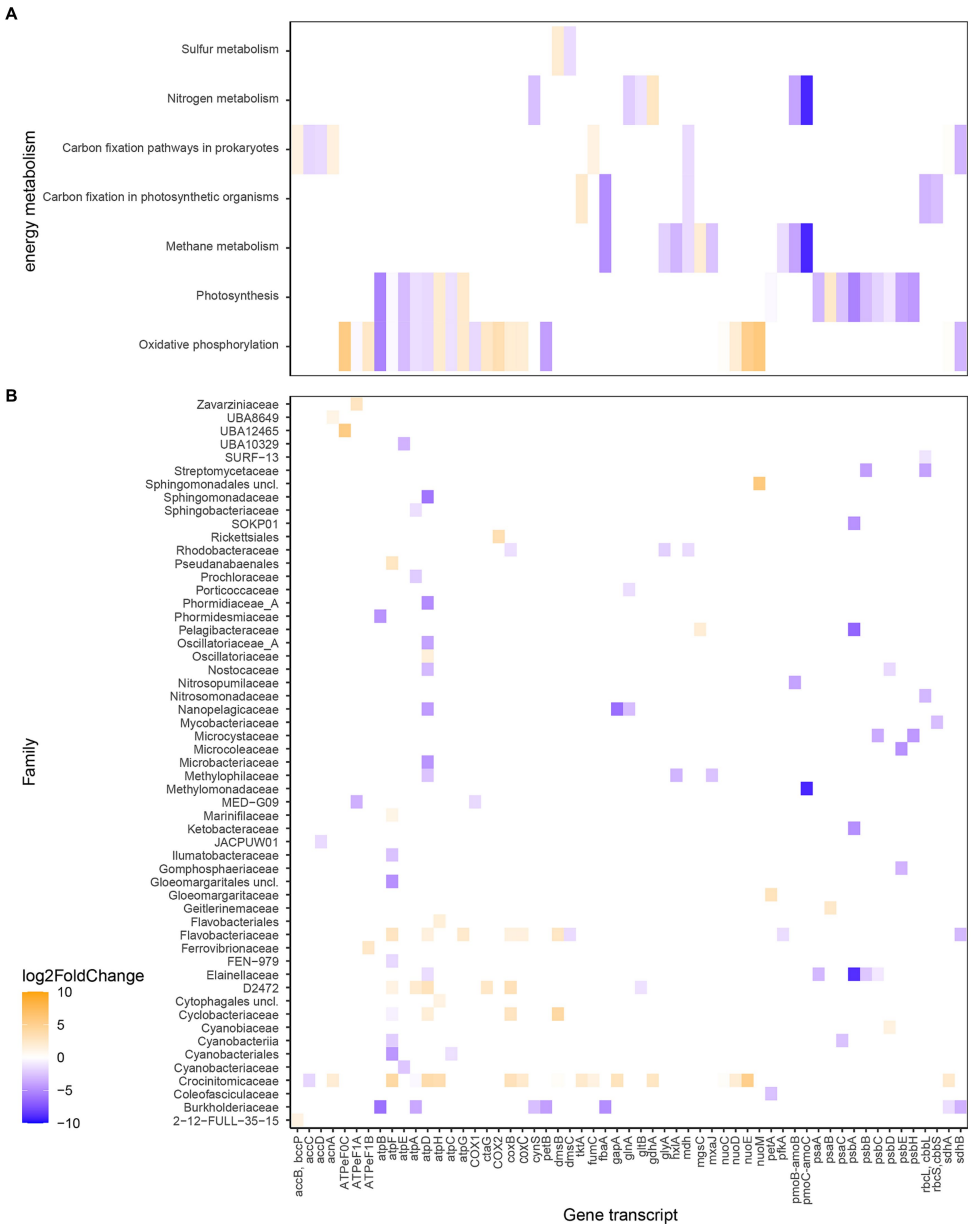
Differential expressed RNA transcript numbers related to energy metabolism according to KEGG Orthology **(A)** and the associated families **(B)**. The blue color (negative values) shows higher RNA transcript numbers (higher Log_2_FoldChange) in the control bay while orange (positive values) shows higher in the heated bay. The *x*-axis shows the genes transcripts (annotated genes), while the *y*-axis shows the different metabolism the gene transcripts are higher expressed in **(A)** and in which taxa they have been higher expressed and associated with **(B)**.

The differences in RNA transcript numbers between the two bays were also shown within gene transcripts related to energy metabolism (Figure 6 and Supplementary Figure S3). Oxidative phosphorylation transcripts showed higher numbers in, e.g., *atpHG* coding for the δ and γ subunits, respectively (Log_2_FoldChange for the whole bay 2.2 and 2.3) in the heated bay compared to *atpAEFB* coding for the α, c, b, and a subunits (−1.8, −3.2, −1.1, and -5.3) in the control bay. This split in ATPase genes between the bays was predominantly along the F_1_ component (subunits γ and δ) in the heated bay attributed to the Flavobacteriaceae family compared to the membrane spanning F_0_ sub-units (c, b, and a) in the control bay from the, e.g., Cyanobiaceae family. Furthermore, genes related to photosynthesis had generally higher transcript numbers in the control bay, such as the photosystem I genes *psaAC* (−3.1 and −2.5) along with the photosystem II genes *psbABCEH* (−5.3, −3.4, −1.9, −3.9, and -4.3) that were for example, attributed to the Elainellaceae, Microcystaceae, and Ketobacteriaceae families. The control bay showed higher transcript numbers of genes related to nitrogen metabolism such as glutamate synthase *gltB* (−1.2) and glutamine synthetase *glnA* (−2.1) both potentially involved in ammonia assimilation by the D2472 and Nanopelagicaceae families (Figure 6).

A noticeable result was the distribution of RNA transcripts attributed to methanogens and methanotrophs in the surface waters of the two bays. For instance, the Log_2_FoldChange values for methane monooxygenase/ammonia monooxygenase genes *pmoC-amoC* (−9.1) and *pmoB-amoB* (−3.8) that based on the transcripts were assigned to the methanotrophic Methylomonadacea (likely *pmoBC*) and the nitrifying (likely *amoBC*) Nitrosopumilaceae families in the control bay (Figure 6 and Supplementary Figure S3). These increased mRNA transcripts were reflected in higher relative abundances of average ASVs within the control bay of methanotrophic microorganisms such as *Methylobacter* (heated 0.04 ± NA control 0.9 ± 0.2% average relative abundance per bay) and *Methyloprofundus* (heated 0.1 ± NA control 0.1 ± 0.003). The opposite pattern was observed with transcripts annotated to genera of archaeal methanogenic organisms that had higher average TPM values in the heated versus control bay such as *Methanobacteriales* (20.8 heated bay), Methanobacteriaceae (10.1 heated bay), and *Methanocullaceae* (17.4 and 4.1).

## Discussion

Long-term climate change related temperature increases lead to a global scale decrease in marine diversity (Colby et al., 2020; Chaudhary et al., 2021). This decrease in diversity is detrimental to community health as a high diversity (combination of different species) buffers against environmental changes (Elmqvist et al., 2003). For instance, an increase in temperature influences the microbial community composition and their energy- and nutrient cycling, which further impacts marine carbon cycling (Abirami et al., 2021; Seidel et al., 2023a). A decline in microbial diversity in the heated bay surface waters of this investigation is supported by previous studies of the heated bay benthic microbial communities (Seidel et al., 2022a).

Large differences were also observed between the heated and control bays for the 16S rRNA gene based microbial communities. For example, higher relative abundances of populations belonging to Proteobacteria and Cyanobacteria were detected in the control bay compared to the heated bay. These shifts in relative abundance between the bays was also shown in previous studies where these phyla showed the highest response to temperature changes (Seidel et al., 2023a). In contrast, Paerl and Huisman (2009) show that for example, Cyanobacteria increase in warming waters and one reason for this discrepancy between the studies could be due to the shift in blooming pattern between the bays with an earlier peak in Cyanobacterial blooms within the heated bay (Seidel et al., 2022a), leading to potentially higher relative abundances in the control bay at the time of the sampling (spring bloom April–May). However, this potential shift in bloom time was only based on observations of four time point measurements for bottom water and surface sediment analyses. Another reason for the difference could be the continuous mixing of the surface waters in the heated bay (due to the influx of warm water from the power plant), reducing the relative abundance of cyanobacterial communities in surface waters (Visser et al., 1996). A shift in spring bloom time could also potentially explain the higher relative abundances of Bacteroidetes in the heated bay, as they are known to be major a responder to phytoplankton blooms (Smith et al., 2017). A second difference in microbial community’s between the bays was higher relative abundance of Flavobacteria in the heated bay that supports the increase in bottom water Flavobacteriales (Seidel et al., 2022a). This was potentially due to the increased abundance of families within this phylum playing a role in organic matter degradation (Bissett et al., 2008; Broman et al., 2019) that is likely increased in the warmer waters.

Microbial community differences between the bays were also demonstrated in, e.g., increased RNA transcripts related to stress in the heated bay as compared to elevated energy related photosynthesis gene transcripts in the control bay. The increase in stress related RNA transcripts within the heated bay has also been observed in surface sediments (Seidel et al., 2022b) and benthic waters (Seidel et al., 2022a). Heat-shock proteins are synthesized within the cell when the ambient temperature is just a few degrees centigrade above the cells optimum (Richter et al., 2010). This supports that the microbial community may not have fully adapted to temperatures in the heated bay after over 50 years of acclimation or alternatively, the stable-state protein synthesis of heat-shock proteins is a part of their adaptation to the warmer surface water [reviewed in Tomanek (2010)]. The increased numbers of control bay photosynthesis related gene transcripts may have been related to the higher relative abundance of Cyanobacteria, which were potentially still in spring bloom state at the time of sampling compared to the heated bay.

Furthermore, a study by Mai et al. (2021) showed that higher temperature leads to a reduction of photosynthetic capacity in phytoplankton in tropical oceans. Another study conducted within the Baltic Sea showed that the cyanobacteria *Rivularia* growth rates decrease with increasing temperatures (Kibria, 2024) while other, e.g., eukaryotic green algae growth increases. This suggests that long-term warmer temperatures can affect Cyanobacteria activity negatively. The data in this study suggested that oxygenic photosynthesis genes had higher transcript numbers in the control bay across different taxa compared to the heated bay while anoxygenic photosynthesis genes were slightly increased in the heated bay. The decreased oxygenic photosynthesis could be a result of the decreased activity of organisms such as Cyanobacteria, as previously described by Bennett et al. (2020) where higher temperatures led to a decrease in oxygenic photosynthesis. The present study could also explain the higher anoxygenic photosynthesis RNA transcripts at warmer temperatures such as resulting from the increased transcripts attributed to Flavobacteria. It has to be mentioned that the mRNA transcript taxonomical annotations were based on the best bitscore, which could result in most likely erroneous assignments such as photosynthesis genes being identified in *Candidatus* Pelagibacter, which could have been likely a contamination of an assembled genome in the database or overlap due to homologous genes, e.g., CO_2_-fixing SAR11 bacteria (Jing et al., 2022). Due to this, these cases of photosynthesis gene mis-annotation were not discussed.

Differences between the two bay’s community structure and RNA transcripts were further underlined by variations in abundances of surface water methanotrophs and methanogens. While there were higher relative abundances as well as transcripts of methanotrophic related microorganisms found in the control bay, higher amounts of methanogenic archaea were found in the surface waters of the heated bay. While the study was carried out in surface waters and both bays were oxic (Supplementary Table S2), these contrasting relative abundances and RNA transcript-based activities might be due to the lower oxygen solubility at raised temperatures favoring anaerobic methanogens (Buan, 2018) versus aerobic methanotrophs (Guerrero-Cruz et al., 2021) in the heated and control bays, respectively. However, it cannot be clarified if these observations were due to potential stratification due to lack of mixing in the control bay or resuspension of sediment due to mixing in the heated bay. Either way, the methanotrophs in the control bay bottom water could lead to methane oxidation while mixing of waters in the heated bay may have resuspended methanogens close to the sediment surface (Seidel et al., 2022b). Alternatively, while methanogenesis most commonly occurs in anoxic conditions, there are records of methane generation in surface waters under oxic conditions in a freshwater lake (Grossart et al., 2011) and in the Baltic Sea (Stawiarski et al., 2019). In general, these findings were in accordance with the discrepancies in microbial community structure between the bays and show that the two bays represented two ecologically contrasting systems.

Two different pore-size filters (0.1 and 0.2 μm) were used without any pre-filtering or a sequential filtration step to capture the surface water microbial communities in the heated and control bays. The lack of significant differences in microbial taxonomical results between the filter pore sizes was likely derived from the filter pores becoming clogged with biomass and particulate matter such that differences between free-living and particle associated microbes could not be discerned. However, the approach removed biases in prokaryotic community structure investigations resulting from sequential filtration of seawater and ensured the whole microbial communities were captured (Padilla et al., 2015).

Finally, it is noteworthy that the variation in alpha diversity within the replicate samples for each bay was high, which could be a result of spatial differences in physical characteristics. Working with natural habitats has many obstacles as even small spatial changes can result in gradients of, e.g., temperature and oxygen concentration creating contrasting environmental niches. Compared to the heated bay, the control bay had a lower salinity, and the control bay salinity was also lower in the surface versus the bottom waters. This indicated an inflow of freshwater in the control bay, as observed in previous studies (Seidel et al., 2022b), and is likely an effect of a freshwater plume from the nearby catchment area to the west. The difference at the time of sampling could be for example due to a predominantly transient or seasonal effect due to snow melt and/or riverine input. Salinity is known to influence bacterial diversity plus community composition (Herlemann et al., 2011) and increases the rate of phytoplankton photosynthesis (Qasim et al., 1972). While the two bays constitute a complex, natural system (that is one of the study’s strengths), the transient shift in salinity was rather small which may also affect microbial populations (Herlemann et al., 2011) but likely not at the observed degree which lead to the conclusion that warmer waters could lead to overall shifts in bacterial community composition over the year and their nutrient cycling. In summary, temperature and its accompanying factors together interplay with location specific environmental conditions to shape microbial communities and result in a complex interlaced relationship that needs to be further investigated to understand future climate change related effects.

## Conclusion

The results of the study added information about how a long-term temperature increase could affect microbial communities and their energy-cycling in coastal surface waters that were in accordance with previous results in benthic and surface sediment microbial communities. However, conclusions must be tentative as the surface water is highly variable, and with different seasonal factors playing key roles for microbial community structure that cannot be supported by a single time-point sample. Hence, sampling over a longer time-period would give a better resolution. With this caveat in mind, the difference in microbial composition and higher stress in the heated bay will likely result in a more fragile ecosystem in the near future.

## Data availability statement

The original contributions presented in the study are included in the article/Supplementary material, further inquiries can be directed to the corresponding author. The R script detailing the analysis can be downloaded at https://github.com/laseab/surf_comp_bay for more detailed information.

## Author contributions

LS: Data curation, Formal analysis, Investigation, Validation, Writing – original draft, Writing – review & editing. EB: Investigation, Validation, Writing – review & editing, Writing – original draft. MS: Investigation, Writing – review & editing, Validation. KB: Investigation, Writing – review & editing, Validation. AF: Conceptualization, Funding acquisition, Validation, Writing – review & editing. SH: Conceptualization, Validation, Writing – review & editing. MK: Conceptualization, Validation, Writing – review & editing. MD: Conceptualization, Funding acquisition, Project administration, Supervision, Writing – original draft, Writing – review & editing.
