# Supplementary material for: Climate change induces shifts in coastal Baltic Sea surface water microorganism stress and photosynthesis gene expression
Source: Front Microbiol. 2024 Jun 7;15:1393538. doi: 10.3389/fmicb.2024.1393538 (PMC11190084; doi:10.3389/fmicb.2024.1393538)
Supplement: Supplementary file 1 [file Data_Sheet_1.docx]

***Supplementary Material***

**Coastal Baltic Sea Surface Water Microorganisms are Affected by Climate Change**

Laura Seidel, Elias Broman, Magnus Ståhle, Kristofer Bergström, Anders Forsman,
Samuel Hylander, Marcelo Ketzer, Mark Dopson

**
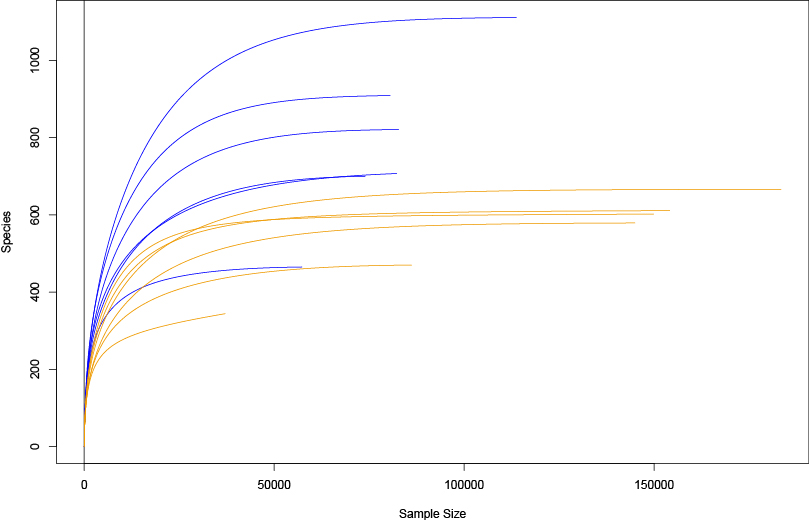
**

**Supplemental Figure 1** **|** Rarefaction curves of the 16S rRNA gene amplicon sequences. The y-axis shows the amount number of DADA2 sequence variants (species) while the x-axis shows the amount of sequence counts (sample size). Sequence counts were rarefied to the lowest sample size for each bay before analysis. The blue lines indicate samples from the control bay, while the orange lines indicate the heated bay.


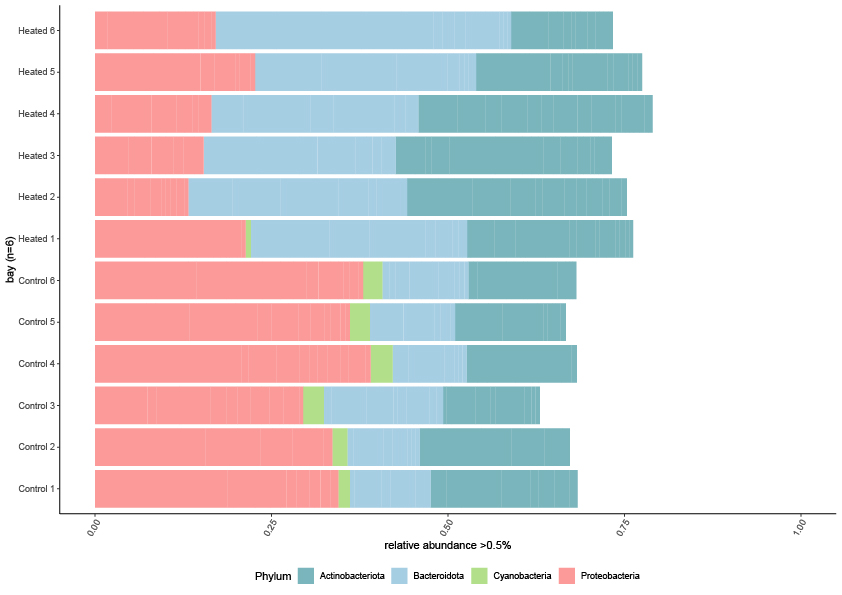


**Supplemental Figure 2 |** Stacked bar plot of 16S rRNA gene amplicon sequencing showing phyla with >0.5 % relative abundance per sample.


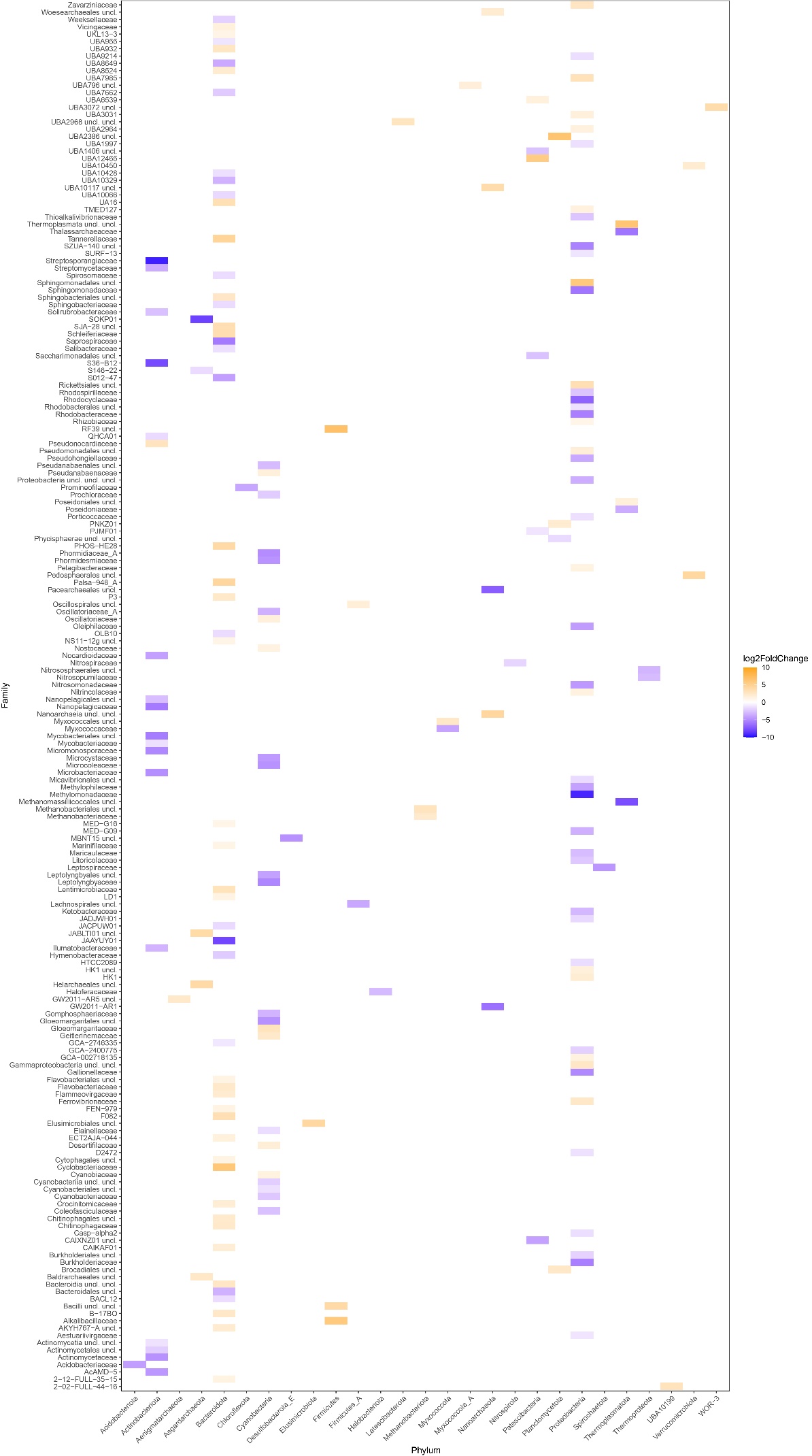


**Supplemental Figure 3 |** Differential expression of transcripts annotated on family and phylum Level. The blue colors indicate higher differential expressed transcripts (higher log2 fold change) in the control bay, while the orange color indicate a higher differential expression in the heated bay.

**Supplemental Table 1.** Overview of measured environmental parameters within the bottom and surface waters of the heated- and control bay at different time points of the year (*n*=5/bay).

| **Parameter** | **month** | **Heated bay** | | **Control bay** | |
| --- | --- | --- | --- | --- | --- |
|  |  | **Surface** | **Bottom** | **Surface** | **Bottom** |
|  |  | **mean±sd** | | | |
| Salinity (‰) | September ´17 | - | - | - | - |
|  | December ´17 ^1^ | 6.8±0 | 6.9±0.28 | 5.8±0.32 | 6.6±0.09 |
|  | April ´18 ^1^ | 6.62±0.04 | 6.6±0.12 | 3.8±0.15 | 6.2±0.74 |
|  | May ´18 | 6.4±0.1 | 6.4±0.1 | 5.4±0.2 | 6.4±0.1 |
|  | June ´18 ^1^ | 6.42±0.04 | 6.4±0.09 | 6.06±0.17 | 6.3±0.08 |
|  | November ´18 | - | - | - | - |
|  | March ´19 | 7.0±0.08 | 6.9±0.13 | 5.2±0.5 | 6.5±0.36 |
| Temperature (°C) | September ´17 ^1^ | 16.6±0.86 | 16.2±0.64 | 15.5±0.21 | 15.2±0.75 |
|  | December ´17 ^1^ | 15.9±0.77 | 12.7±1.93 | 3.8±1.08 | 4.0±1.51 |
|  | April ´18 ^1^ | 13.8±1.13 | 13.1±0.66 | 10.1±0.77 | 7.0±1.58 |
|  | May ´18 | 20.9±0.75 | 20.6±0.26 | 17.7±0.2 | 14.3±1.9 |
|  | June ´18 ^1^ | 20.4±1.08 | 19.9±2.04 | 14.8±1.28 | 12.1±1.60 |
|  | November ´18 | 15.2±1.77 | 14.9±1.25 | 8.6±1.05 | 9.7±2.45 |
|  | March ´19 | 11.6±1.96 | 11±0.52 | 5.9±0.45 | 5.6±0.40 |

^1^partially published data <https://doi.org/10.3389/fmicb.2022.873281>

**Supplemental Table 2.** Details of the 16S rRNA gene (top) and metatranscriptome (bottom) sequencing.

16S rRNA gene

| **Bioproject** | **Sample** | **Replicate** | **Date** | **Temp** | **pH** | **Salinity** | **O_2_** | **Site** | **Filter** | **Bay** | **Sequences** | **Filtered** | **Remaining** | **DADA2** | **Final** |
| --- | --- | --- | --- | --- | --- | --- | --- | --- | --- | --- | --- | --- | --- | --- | --- |
|  |  |  |  | **°C** |  | **‰** | **mg/L** |  | **µm** |  |  | **sequences** | **sequences (%)** | **input** | **sequences** |
| SRX5362130 | X1083 | 1 | 17 Apr 2018 | 10.4 | 7.93 | 3.8 | 15.7 | K | 0.1 | Control | 169,392 | 168,895 | 99.70 | 168871 | 120063 |
| SRX5362131 | X1084 | 2 | 17 Apr 2018 | 10.4 | 7.93 | 3.8 | 15.7 | K | 0.1 | Control | 137,371 | 137,035 | 99.80 | 137005 | 88687 |
| SRX5362132 | X1085 | 3 | 17 Apr 2018 | 10.4 | 7.93 | 3.8 | 15.7 | K | 0.1 | Control | 134,352 | 134,034 | 99.80 | 133995 | 89211 |
| SRX5362133 | X1086 | 1 | 17 Apr 2018 | 10.4 | 7.93 | 3.8 | 15.7 | K | 0.2 | Control | 129,06 | 128,739 | 99.80 | 128711 | 78325 |
| SRX5362134 | X1087 | 2 | 17 Apr 2018 | 10.4 | 7.93 | 3.8 | 15.7 | K | 0.2 | Control | 164,68 | 164,239 | 99.70 | 164212 | 88282 |
| SRX5362135 | X1088 | 3 | 17 Apr 2018 | 10.4 | 7.93 | 3.8 | 15.7 | K | 0.2 | Control | 114,427 | 114,141 | 99.80 | 114124 | 61943 |
| SRX5362136 | X1089 | 1 | 16 Apr 2018 | 13.6 | 8.4 | 6.6 | 14.6 | C | 0.1 | Heated | 114,709 | 114,368 | 99.70 | 114354 | 37277 |
| SRX5362137 | X1090 | 2 | 16 Apr 2018 | 13.6 | 8.4 | 6.6 | 14.6 | C | 0.1 | Heated | 235,609 | 234,95 | 99.70 | 234906 | 184777 |
| SRX5362138 | X1091 | 3 | 16 Apr 2018 | 13.6 | 8.4 | 6.6 | 14.6 | C | 0.1 | Heated | 210,943 | 210,416 | 99.80 | 210373 | 151826 |
| SRX5362139 | X1092 | 1 | 16 Apr 2018 | 13.6 | 8.4 | 6.6 | 14.6 | C | 0.2 | Heated | 204,585 | 204,088 | 99.80 | 204067 | 146523 |
| SRX5362128 | X1093 | 2 | 16 Apr 2018 | 13.6 | 8.4 | 6.6 | 14.6 | C | 0.2 | Heated | 158,322 | 157,921 | 99.70 | 157902 | 87417 |
| SRX5362129 | X1094 | 3 | 16 Apr 2018 | 13.6 | 8.4 | 6.6 | 14.6 | C | 0.2 | Heated | 221,45 | 220,943 | 99.80 | 220885 | 157924 |

RNA transcripts

| **Bioproject** | **Sample** | **Replicate** | **Date** | **Site** | **Filter** | **Bay** | **Trimmed** | **Non-** | **Mapped** | **Unmapped** | **Feature** | **Prokka** | **Eggnog** | **Kofamscan** |
| --- | --- | --- | --- | --- | --- | --- | --- | --- | --- | --- | --- | --- | --- | --- |
|  |  |  |  |  | **µm pore** |  | **sequences** | **contaminated** | **sequences** | **sequences** | **count** | **orfs** | **orfs** | **orfs** |
| SRX4994487 | 101 | 1 | 17 April 2018 | K | 0.1 | Control | 158960888 | 10686814 | 7225285 | 103915 | 1664623 | 16210 | 16377 | 19206 |
| SRX4994486 | 102 | 2 | 17 April 2018 | K | 0.1 | Control | 135286304 | 4820188 | 2772230 | 66170 | 494646 | 12977 | 12909 | 14557 |
| SRX4994489 | 103 | 3 | 17 April 2018 | K | 0.1 | Control | 103760992 | 5850910 | 3663947 | 60527 | 781317 | 14374 | 14447 | 16646 |
| SRX4994488 | 104 | 1 | 17 April 2018 | K | 0.2 | Control | 76564500 | 4716056 | 3042482 | 44280 | 687620 | 10646 | 11122 | 13033 |
| SRX4994491 | 105 | 2 | 17 April 2018 | K | 0.2 | Control | 114169948 | 8257556 | 5747538 | 76308 | 1378723 | 12909 | 13230 | 15694 |
| SRX4994490 | 106 | 3 | 16 April 2018 | C | 0.1 | Heated | 157802858 | 10520708 | 7726927 | 85247 | 1295004 | 18913 | 18927 | 21961 |
| SRX4994493 | 107 | 1 | 16 April 2018 | C | 0.1 | Heated | 63720406 | 5339360 | 3973171 | 40047 | 600837 | 16520 | 16418 | 18920 |
| SRX4994492 | 108 | 2 | 16 April 2018 | C | 0.1 | Heated | 68680808 | 8677594 | 6867823 | 60675 | 1110821 | 17539 | 17044 | 19362 |
| SRX4994485 | 109 | 3 | 16 April 2018 | C | 0.2 | Heated | 174257488 | 13485120 | 11109837 | 89787 | 2238155 | 14217 | 14247 | 16101 |
| SRX4994484 | 110 | 1 | 16 April 2018 | C | 0.2 | Heated | 86336396 | 7973062 | 6453906 | 62464 | 1257780 | 16288 | 16122 | 18492 |
| SRX4994494 | 111 | 2 | 16 April 2018 | C | 0.2 | Heated | 41231490 | 2998106 | 2140887 | 23177 | 388497 | 12592 | 12386 | 14058 |

**Supplemental Table 3.** PERMANOVA on 16S rRNA gene amplicon dataset (16S) and transcriptome mRNA dataset (mRNA) to test for differences between filter pore sizes. The option “strata” was used to exclude the effect of bay; permutations=999.

| **Variable** | **Df** | **SumsofSqs** | **F-Model** | ***p*-value** |
| --- | --- | --- | --- | --- |
| Filter (16S) | 1 | 0.02 | 0.11 | 0.787 |
| Filter (mRNA) | 1 | 0.11 | 0.49 | 0.392 |
